# Dataset on unmanned aerial vehicle multispectral images acquired over a vineyard affected by *Botrytis cinerea* in northern Spain

**DOI:** 10.1016/j.dib.2022.108876

**Published:** 2023-01-02

**Authors:** Sergio Vélez, Mar Ariza-Sentís, João Valente

**Affiliations:** Information Technology Group, Wageningen University & Research, Wageningen 6708 PB, Netherlands

**Keywords:** Viticulture, Precision agriculture, Disease detection, UAV, Drone, Orthomosaic, Structure from motion, Photogrammetry

## Abstract

Remote sensing makes it possible to gather data rapidly, precisely, accurately, and non-destructively, allowing it to assess grapevines accurately in near real-time. In addition, multispectral cameras capture information in different bands, which can be combined to generate vegetation indices useful in precision agriculture. This dataset contains 16,504 multispectral images from a 1.06 ha vineyard affected by *Botrytis cinerea*, in the north of Spain. The photos were taken throughout four UAV flights at 30 m height with varying camera angles on 16 September 2021, the same date as the grape harvest. The first flight took place with the camera tilted at 0° (nadir angle), the second flight at 30°, the third flight at 45°, and the fourth flight was also performed at 0° but was scheduled in the afternoon to capture the shadows of the plants projected on the ground. This dataset was created to support researchers interested in disease detection and, in general, UAV remote sensing in vineyards and other woody crops. Moreover, it allows digital photogrammetry and 3D reconstruction in the context of precision agriculture, enabling the study of the effect of different tilt angles on the 3D reconstruction of the vineyard and the generation of orthomosaics.

**Specifications Table**

**Table:** 

Subject	Agricultural Sciences, Agronomy and Crop Science
Specific subject area	Disease detection in Precision Agriculture using UAVs
Type of data	Image
How the data were acquired	Aerial Platform: DJI M210 multi-rotor platform GNSS RTK Rover device: Trimble R2 Integrated GNSS system with a TSC3 Controller Flight speed: 2 m/s Overlap: 80% Flight altitude: 30 m AGL Sensor: Micasense RedEdge 3 multispectral camera Sensor characteristics: Focal aperture: f2.8, Shutter speed: 1/125, Interval shooting time: 1 s, Aperture: f/2.8, Exposure time: 1/523 s Four flights were carried out: (1) 0_V1 (30 m height, 0°) (2) 30_V1 (30 m height, 30°) (3) 45_V1 (30 m height, 45°) (4) 0_V2 (30 m height, 0° (afternoon))
Data format	Raw
Description of data collection	UAV flights took place on 16 September 2021, the same date as the grape harvest, at 30 m height and using different angles. The 4th flight was scheduled in the afternoon to capture the shadows of the plants following Vélez et al. (2021) method. In addition, pictures of the provided Micasense calibrated reflectance panel were captured from directly overhead it. The flight path was programmed to fly autonomously, following the manufacturer's instructions (DJI). The dataset includes a shapefile with the GPS location of vine trunks, grape clusters affected by Botrytis and GCPs.
Data source location	Institution: Wageningen University & Research City/Town/Region: Tomiño, Pontevedra, Galicia Country: Spain Latitude and longitude (and GPS coordinates) for collected samples/data: 41°57′18.5″N 8°47′41.2″W
Data accessibility	Repository name: Zenodo Data identification number: https://doi.org/10.5281/zenodo.7064894 Direct URL to data: https://zenodo.org/record/7383601
Related research article	Vélez, S.; Ariza-Sentís, M.; Valente, J. Mapping the Spatial Variability of Botrytis Bunch Rot Risk in Vineyards Using UAV Multispectral Imagery. European Journal of Agronomy 2023, 142, 126691, doi:10.1016/j.eja.2022.126691.

**Value of the Data**

• Data is useful for researchers interested in UAV (unmanned aerial vehicle) remote sensing in vineyards and other woody crops. Moreover, it allows digital photogrammetry and 3D reconstruction in the context of precision agriculture.
• Dataset allows studying the effect of using different tilt angles on the 3D reconstruction of the vineyard and the generation of orthomosaics.
• Dataset can be employed to develop new vegetation indices and algorithms for disease detection in vineyards [1].
• Dataset can be used to study the relationship between the spectral information of the vegetation and the plant health status.
• Dataset can be utilized as a resource for image segmentation and allows the development of new techniques for trunk detection, plant isolation and vegetation segmentation in agriculture.
• Dataset can be employed to build multispectral dense clouds and obtain more information than in a single orthomosaic.

## 1 Objective

The objective of this dataset is to gather multispectral images of the vineyard with varying conditions to ensure enough variability (1) to study the effect of changing imaging parameters on vegetation segmentation and identification, (2) to allow the detection of *Botrytis cinerea* in vineyards using UAV imagery, and (3) for studies aiming to conduct individual grapevine identification. For this purpose, four flights were made at different times of the day and with different camera tilt angles. These multispectral images allow the generation of orthomosaics and vegetation indices and the 3D reconstruction of the vineyard.

## 2 Data Description

**Fig. 1:**
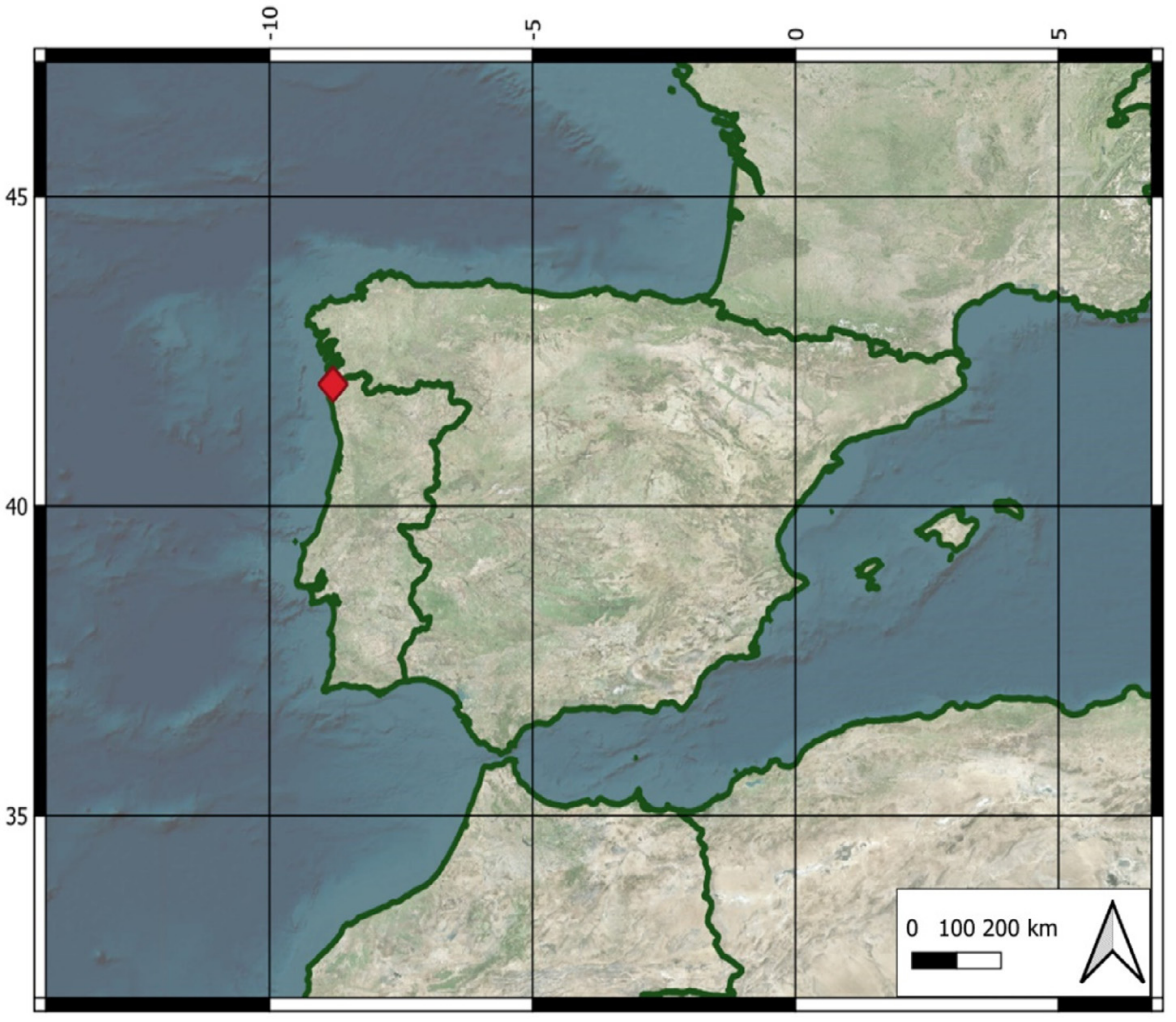
Vineyard location (red) in Tomiño, Pontevedra, Spain. GPS coordinates: 41°57′18.5″N 8°47′41.2″W.

This work describes a set of ground data and four flights captured on grape harvest (16 September 2021), using a Trimble R2 Integrated GNSS system with a TSC3 Controller, a commercial UAV (a DJI M210 multi-rotor platform UAV) and a multispectral sensor (Micasense Rededge 3) over a commercial vineyard (41°57′18.5"N 8°47′41.2"W) property of 'Bodegas Terras Gauda S.A.', located in Tomiño, Pontevedra, within the region of Galicia, Spain (Fig. 1). Plants were grafted onto 196.17C rootstocks, tolerant to activated limestone, adapted to soils with excess moisture, and trained in vertical shooting positioning (VSP). The vineyard was planted in 1990 with a NE-SW orientation, a plant-to-plant distance of 2.5 m and a distance between rows of 3 m. Spontaneous vegetation species grew as an intercrop.

### 2.1 UAV Multispectral Data

**Fig. 2:**
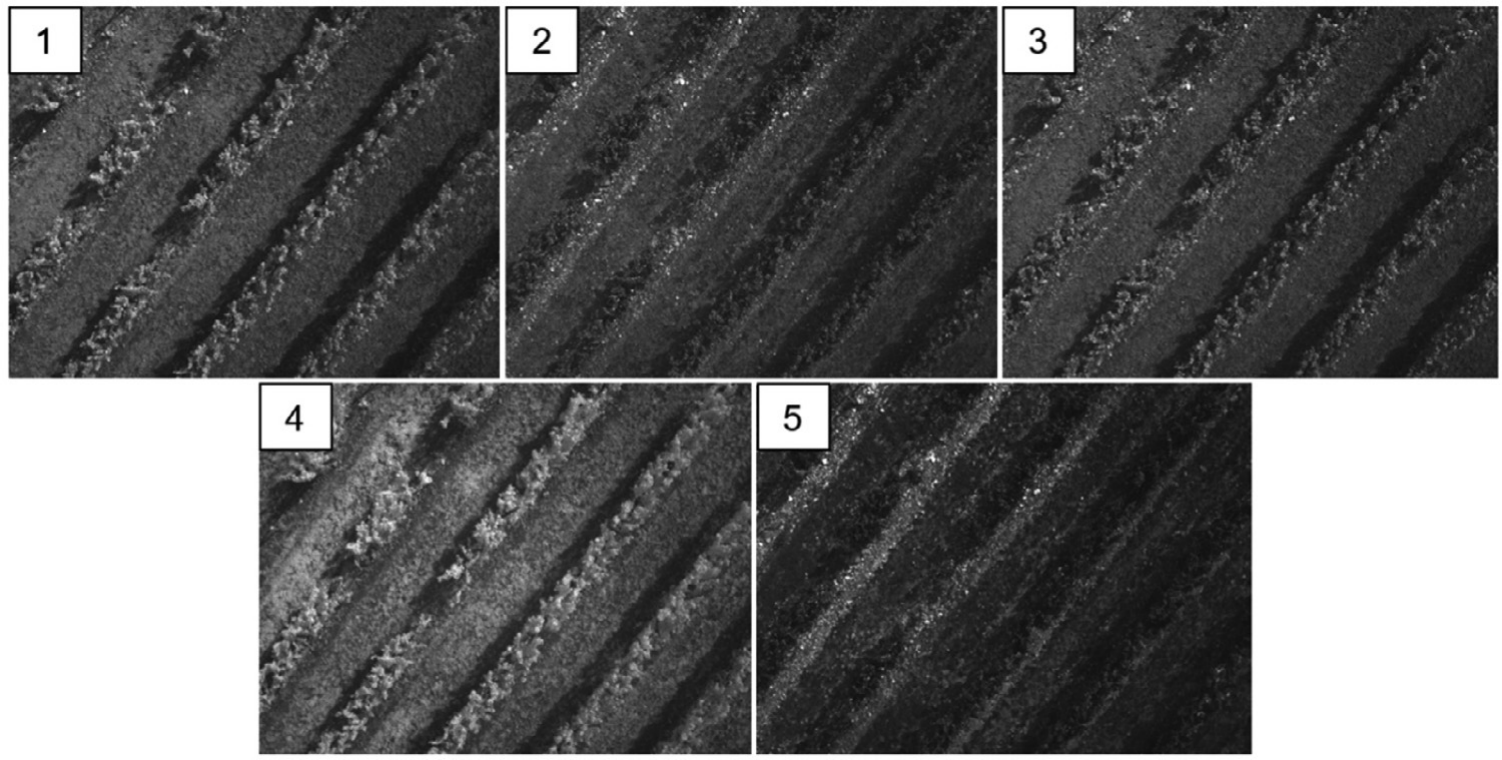
Multispectral imagery taken at 30 m height. Each shot captures five bands: 1: blue, 2: green, 3: red, 4: near-infrared and 5: red edge, in separated tif files.

This dataset is composed of a total of 16,504 multispectral images. Table 1 shows the number of aerial photographs taken per flight. Each shot of the Micasense Rededge 3 multispectral camera captures five bands (blue, green, red, red edge and near-infrared) in separated tif (Tagged Image Format) files (Fig. 2). Table 2 shows the band number, name, center and wavelength (nm) of each band, according to the specifications provided by the manufacturer. The image names are structured as ``IMG_imageNumber_bandNumber''. For example, ``IMG_0801_1'' is band 1 (blue) of image number 801 of the flight.

**Table 1:** Number of images taken per flight. ``Panel'' are the images of the Micasense calibrated reflectance panel, and ``Set'' are the images over the vineyard. Total images: 16,504.

Flight number	Flight name	Description	Panel	Set
1	0_V1	30 m height, 0°	65	6355
2	30_V1	30 m height, 30°	45	2630
3	45_V1	30 m height, 45°	40	4354
4	0_V2	30 m height, 0° (afternoon)	20	2995

**Table 2:** Band number, name, center and wavelength (nm) of each Micasense Rededge 3 multispectral camera band.

Band number	Band name	Center	Bandwidth
1	Blue	475 nm	20 nm
2	Green	560 nm	20 nm
3	Red	668 nm	10 nm
4	Near-infrared	840 nm	40 nm
5	Red edge	717 nm	10 nm

### 2.2 Ground-truth data

**Fig. 3:**
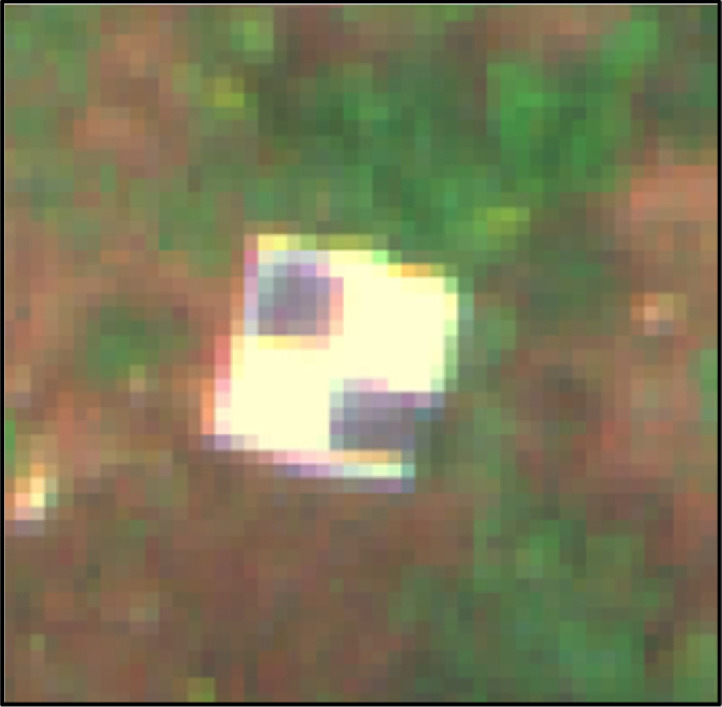
Ground Control Point (GCP), which can be identified as a black and white tile in the images.

The shapefile includes two types of points: (1) Ground Control Points (GCPs), which in the images can be discerned as black and white tiles on the ground (Fig. 3); and (2) The location of each Botrytis bunch rot infected cluster. Both layers are projected in ETRS89/UTM zone 29N CRS (coordinate reference system).

## 3 Experimental Design, Materials and Methods

The flights were performed on 16 September 2021, the same date as the grape harvest, over a 1.06 ha and 8.1% slope vineyard, *Vitis vinifera* cv. Loureiro.

### 3.1 UAV multispectral data

**Fig. 4:**
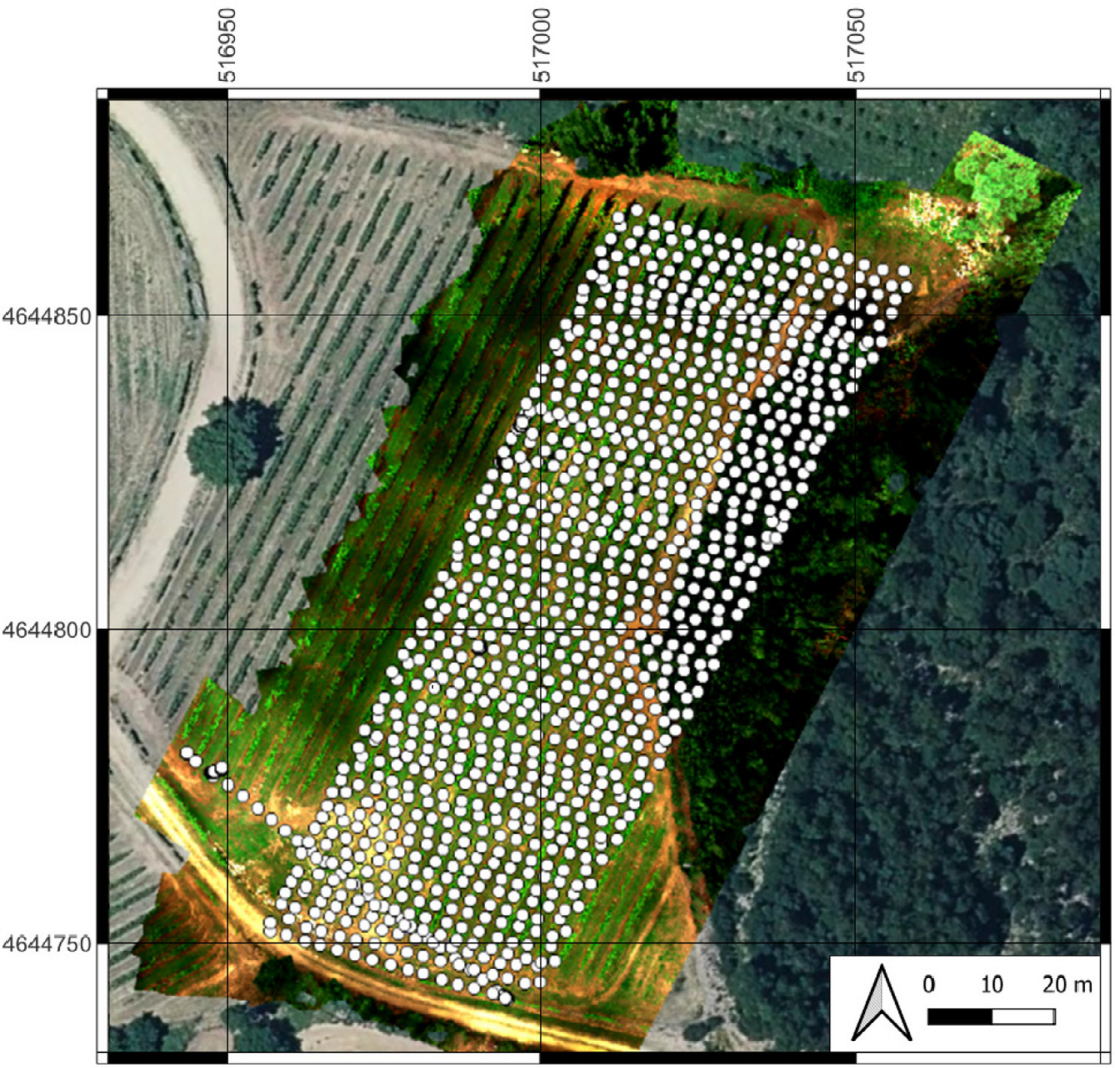
Location of the geotagged images. White dots are the location of the photos over the vineyard in the first flight (0° – nadir angle). The background Orthomosaic (not included) was created using the RAW images provided in this dataset. Flight altitude: 30 m.

Four UAV flights at 30 m height were performed using different camera angles. The first flight took place with the camera tilted at 0°; the second flight at 30°; the third flight at 45°, and the fourth flight was also performed at 0° but scheduled in the afternoon to capture the shadows of the plants following Vélez method [2]. The planned overlap was 80%. Fig. 4 shows the location of the images over the vineyard (white dots) in the first flight (0° – nadir angle). Pictures of the provided Micasense calibrated reflectance panel were captured after each flight. The flight path was programmed to fly autonomously, following the manufacturer's instructions (DJI). The mission was planned using the official 'DJI Pilot' app to ensure a safe flight and enough overlap coverage. Flight conditions during the aerial survey were clear sky, with some isolated clouds, and wind velocity of less than 0.5 m/s. The Micasense Rededge 3 sensor has a 4.8 mm x 3.6 mm size, with a 3.75 μm pixel size, and 1280 × 960 resolution for each band. In addition, other characteristics are focal aperture: f2.8, shutter speed: 1/125, interval shooting time: 1 s, aperture: f/2.8, and exposure time: 1/523 s. All images were geotagged automatically by the Micasense camera in EXIF format, capturing the data in a Lat/Long coordinate system (WGS84). In order to extend the possibilities of the dataset for the researchers (e.g. to develop software to filter out/eliminate useless images automatically), the images were not modified or filtered; they are ``as is'', i.e. as taken by the multispectral sensor, including images acquired before and/or after the UAV starts the flight plan itself.

### 3.2 Ground-Truth Data

Seven GCPs were taken to enhance potential 3D reconstruction and mosaicking and, therefore, increase the spatial accuracy of the orthomosaic [3]. Likewise, every grape cluster affected by botrytis bunch rot was located, focusing on three specific vineyard rows. In addition, the location of each vine trunk was marked. The locations were taken using a Trimble R2 Integrated GNSS system with a TSC3 Controller (Trimble Inc., California, USA) that provides centimetre positioning accuracy. Finally, the disease was identified according to the literature [4], and the threshold for determining botrytis disease infection (botrytis bunch rot) was set following the guidelines of the European and Mediterranean Plant Protection Organization [5], designating the 'presence of botrytis disease' as 'positive' from EPPO scale levels 2 to 5 and 'negative' for EPPO scale level 1.

## Ethics Statements

The authors state that the present work meets the ethical requirements for publication in Data in Brief. The work does not involve studies with animals and humans.

## CRediT authorship contribution statement

**Sergio Vélez:** Investigation, Methodology, Data curation, Writing – original draft. **Mar Ariza-Sentís:** Visualization, Writing – review & editing, Methodology, Data curation. **João Valente:** Conceptualization, Supervision, Writing – review & editing.

## Declaration of Competing Interest

The authors declare that they have no known competing financial interests or personal relationships that could have appeared to influence the work reported in this paper.

## Data Availability

UAV multispectral imagery dataset over a vineyard affected by Botrytis in `Tomiño', Pontevedra, Spain. Includes GPS location of diseases and GCP points (Original data) (Zenodo).
